# Correlation of image quality parameters with tube voltage in X-ray dark-field chest radiography: a phantom study

**DOI:** 10.1038/s41598-021-93716-5

**Published:** 2021-07-08

**Authors:** Andreas P. Sauter, Jana Andrejewski, Manuela Frank, Konstantin Willer, Julia Herzen, Felix Meurer, Alexander A. Fingerle, Markus R. Makowski, Franz Pfeiffer, Daniela Pfeiffer

**Affiliations:** 1 Department of Diagnostic and Interventional Radiology, School of Medicine and Klinikum Rechts Der Isar, Technical University of Munich, Ismaningerstr. 22, 81675 Munich, Germany; 2 Chair of Biomedical Physics, Department of Physics and Munich School of BioEngineering, Technical University of Munich, Garching, Germany

**Keywords:** Techniques and instrumentation, Medical research, Respiratory signs and symptoms

## Abstract

Grating-based X-ray dark-field imaging is a novel imaging modality with enormous technical progress during the last years. It enables the detection of microstructure impairment as in the healthy lung a strong dark-field signal is present due to the high number of air-tissue interfaces. Using the experience from setups for animal imaging, first studies with a human cadaver could be performed recently. Subsequently, the first dark-field scanner for in-vivo chest imaging of humans was developed. In the current study, the optimal tube voltage for dark-field radiography of the thorax in this setup was examined using an anthropomorphic chest phantom. Tube voltages of 50–125 kVp were used while maintaining a constant dose-area-product. The resulting dark-field and attenuation radiographs were evaluated in a reader study as well as objectively in terms of contrast-to-noise ratio and signal strength. We found that the optimum tube voltage for dark-field imaging is 70 kVp as here the most favorable combination of image quality, signal strength, and sharpness is present. At this voltage, a high image quality was perceived in the reader study also for attenuation radiographs, which should be sufficient for routine imaging. The results of this study are fundamental for upcoming patient studies with living humans.

## Introduction

Grating-based X-ray imaging is an emerging imaging modality, visualizing dark-field, phase-contrast, and conventional attenuation information with the same acquisition^1^. The dark-field modality shows small-angle scattering whereas the phase-contrast modality reflects the magnitude of X-ray refraction, respectively. An optical grating induces a high frequency intensity pattern on the beam at certain distances, the so called “fractional Talbot distances”. The relative contrast resulting from this pattern is called “interferometric visibility”. Attenuation, refraction, and small-angle scatter induced by an object in the beam path modify the intensity pattern as follows: X-ray attenuation reduces the mean value, refraction induces a lateral shift and small angle scattering reduces the visibility of this pattern. The dark-field image can be calculated from the ratio of reduced and original visibility without a sample in the beam path. As the pattern is typically in the order of a few micrometers, a second grating is placed in front of the detector to allow implementation with common medical X-ray detectors with larger pixel sizes. An additional third grating near the X-ray source allows exploiting the Lau effect, enabling the use of the setup with conventional X-ray sources. Such an arrangement of 3 gratings allows the usage of common medical X-ray equipment and thus enables the application in a clinical surrounding^2,3^.

Small-angle X-ray scattering occurs when a high number of interfaces between structures with a different refractive index are present, e.g. at air-tissue interfaces of alveoli in the lung or at bone-fat interfaces in spongy bone. This results in a high dark-field signal generated by these tissues. In contrast, conventional X-ray imaging detects only the degree of X-ray attenuation. Thus, dark-field imaging provides a completely new and complementary image information. Numerous applications for dark-field and phase-contrast imaging have been described as results of technical studies as well as of in-vivo and ex-vivo animal studies^4–10^. Additionally, real-time, dynamic imaging of lung function as well as the visualization of liquid-filled airways is possible with phase-contrast imaging as a study in rabbits could show^9^. Besides the lung as one important field of this technique, soft-tissues such as the brain can be visualized via phase-contrast imaging^10^. Dark-field imaging of the lung is of a high interest as in the healthy lung a strong dark-field is present due to the very high number of air-tissue interfaces^11^. Lung diseases reducing the number of air-tissue interfaces also reduce the dark-field signal, i.e. pulmonary fibrosis or pulmonary emphysema. Earlier animal studies showed that dark-field imaging enables the detection of pulmonary fibrosis and pulmonary emphysema at early stages with sensitivities distinctively increased compared to conventional radiographs^8,12^. Recently reported results from in-vivo pig or postmortem human cadaver studies demonstrated the implementation of dark-field chest imaging within clinical boundary conditions with regards on scan time, field of view, and radiation dose^5,13–15^.

X-Ray (CXR) is a common first imaging modality of the chest as it is widely available, relatively cheap, and fast. Only a low radiation dose of 0.02 mSv (posteroanterior) or 0.1 mSv (posteroanterior and lateral combined) is reported for this modality^16^. This radiation dose is similar to the dose which is maximally allowed by the local authorities for the setup used in the current study which is 0.15 mSv for posteroanterior and lateral imaging combined.

For X-ray imaging, multiple studies examining the optimal tube voltages (kVp) for projection radiographs exist^17,18^. Despite a tube voltage of 110–130 kVp being used in clinical routine and favored in some studies^17^, another study suggests a tube voltage of 90 kVp as they reported an optimum overall image quality at this lowest tested voltage^18^. As the fraction share of the Compton scattering increases and the share of the photoelectric effect decreases with higher tube voltages, the relative absorption of bone in chest radiographs is higher at lower tube voltages. As a result, in clinical routine, conventional projection radiographs of the lung are obtained using 120–130 kVp, whereas for skeletal radiographs a tube voltage of 70 to 80 kVp is used^19,20^. An important consideration for attenuation and dark-field X-ray imaging is the tradeoff between image quality and patient dose, according to the principle of “as low as reasonably achievable”. Amongst others, both depend on the tube voltage. For dark-field imaging, the additional limitation exists that both dark-field signal generated by the imaged organ and interferometric visibility should be sufficiently high. The magnitude of the dark-field signal decreases with increasing energy $E$, as the decrement $\delta$ of the real part of the complex refractive index decreases with $E^{2}$^21^. The setup's autocorrelation length $\xi$ and sample autocorrelation function also influence the energy dependence of the dark-field signal, as an increase of $E$ results in a decrease of $\xi$^22,23^. This leads to a further decrease of the dark-field signal for increasing energies if other setup parameters such as the period of the modulation grating, or the object position remain unchanged. To achieve a relatively good interferometric visibility for high energies, either modulation gratings with small periods, while the setup length and Talbot order remain constant, or long setup lengths, while the grating periods and Talbot order remain the same, are needed. However, for both parameters exist technical and spatial constraints. In LIGA manufactured absorption gratings, gold is often used as an absorber material due to its strong absorbing properties^24^. However, producing high and strongly absorbing gratings with low periods is technically difficult. Just below the K-edge of gold at 80.7 keV, the grating is quite transparent for X-rays and the interferometric visibility is almost zero. Furthermore, the setup should fit into a laboratory or clinical examination room and further technical complications might occur for long setups.

Due to the described physical requirements, generally dark-field imaging is performed with lower tube voltages. An earlier study examined signal strength and image quality of dark-field images of an in-situ human lung at different tube voltages^6^. In that study, optimal tube voltages of 60–70 kVp were proposed. However, this study was performed in an experimental setup. The usage of a deceased body enabled the examination of a real human lung, however it had to be inflated and infiltrates were present. Additionally, the patient dose was not kept constant which could lead to distorted results due to variations in image noise. The aim of the current study was to determine the optimal tube voltage for dark-field radiographs in a setup suitable for studies of living humans using a lung phantom mimicking shape and X-ray interaction of a human thorax. Additionally, the attenuation radiographs of the respective tube voltages were evaluated.

## Results

**Figure 1:**
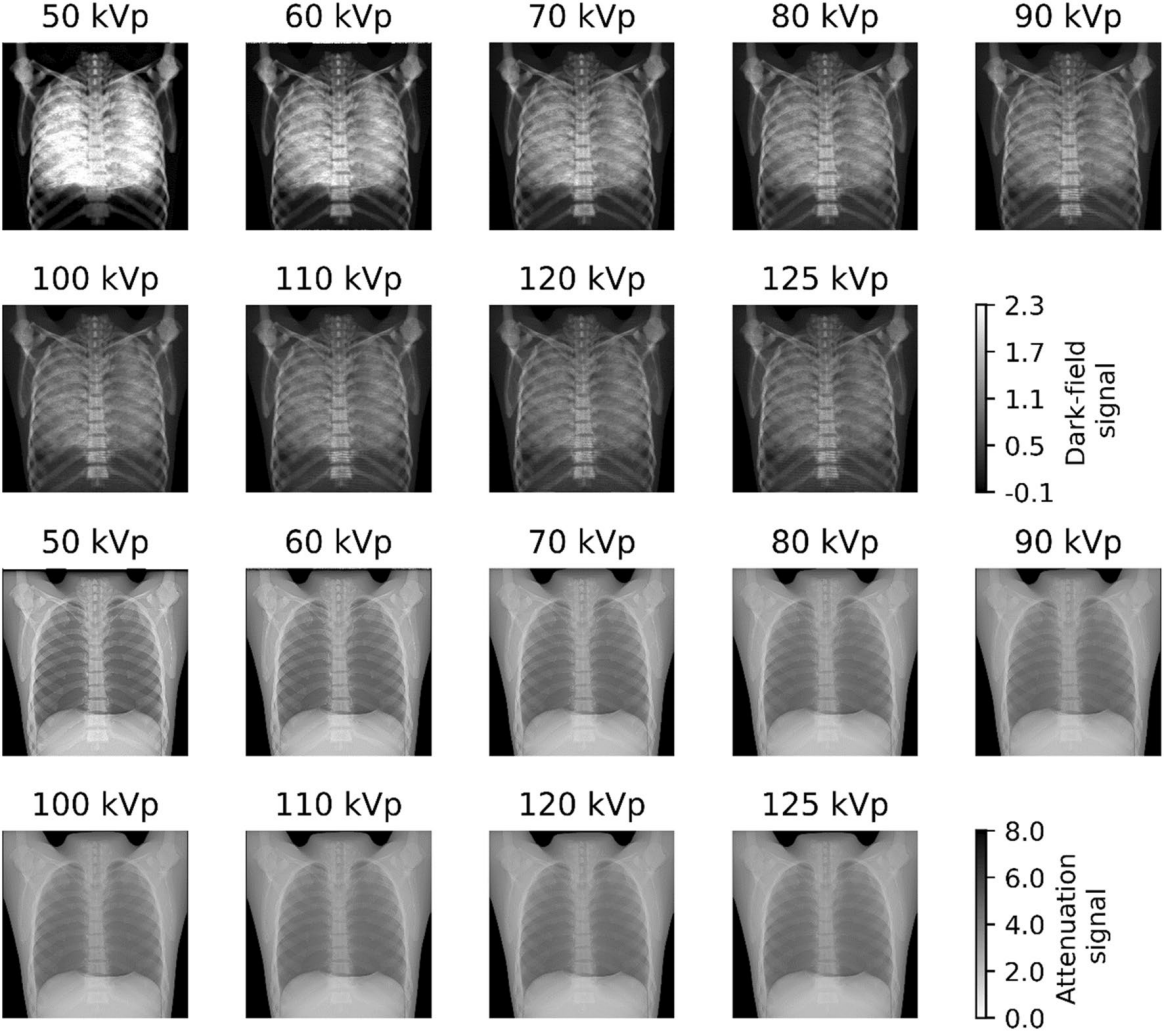
Dark-field (top) and attenuation radiograph (bottom) images acquired with different tube voltages. A distinct decrease in dark-field signal strength can be seen towards higher tube voltages. At lower tube voltages, a higher opacity of bone is present in attenuation radiographs, impairing the visualization of the “lung”.

The acquired dark-field and attenuation radiographs are shown in Fig. 1.

### Reader study

**Figure 2:**
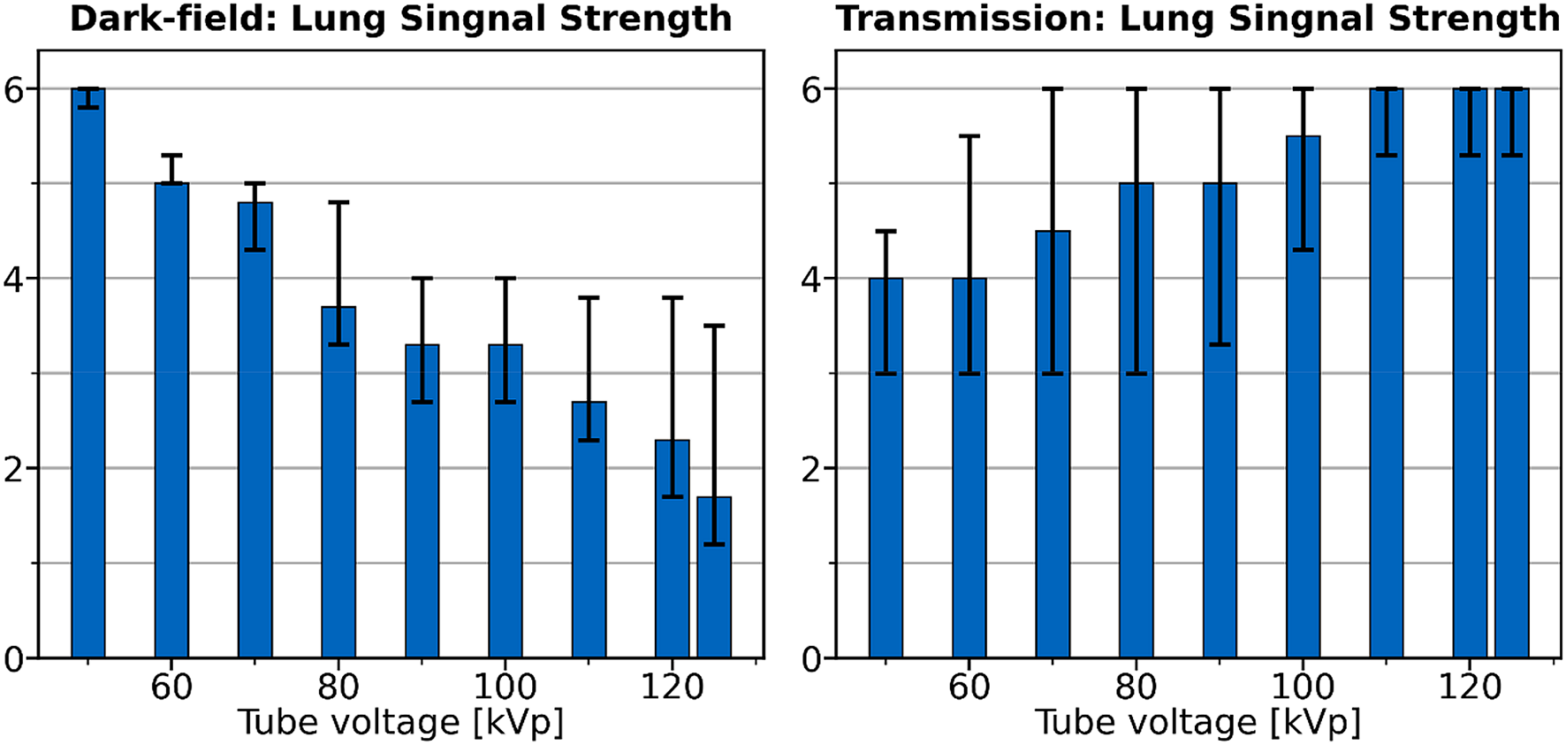
Dark-field and attenuation signal strength rating in the reader study. Given is the median (bars: range) rating of the readers after calculating the mean for all areas of the lung and both reading sessions for each reader. For dark-field images, the signal strength of the lung was rated highest at 50 kVp and decreased towards higher tube voltages. Additionally, it was clearly higher at 60 kVp and 70 kVp compared to higher voltages with medians of 5.0 and 4.8, respectively. For attenuation images, the subjective lung signal was higher at higher tube voltages with a median 6.0 for 110–125 kVp, respectively. Despite the clear trends, no significant differences (Wilcoxon-signed rank test) for the signal strength at different voltages could be found.

Dark-field signal strength showed the highest rating at 50 kVp with a median value of 6.0 (Fig. 2).

For dark-field images, the highest image quality could be found for 70 kVp and 80 kVp (Table 1). The sharpness of the dark-field images was rated highest for 50 to 70 kVp. These results were not statistically significant (Wilcoxon-signed rank test).

**Table 1:** Results of the reader study examining the dark-field images.

	Lung signal	Bone overlay	Image quality	Sharpness
50 kVp	6.0 (5.8–6.0)	6.0 (4.8–6.0)	5.5 (4.5–6.0)	5.5 (2.0–6.0)
60 kVp	5.0 (5.0–5.3)	5.5 (4.8–6.0)	5.0 (4.5–6.0)	5.5 (2.0–5.5)
70 kVp	4.8 (4.3–5.0)	5.5 (4.0–5.5)	6.0 (5.0–6.0)	5.5 (4.0–6.0)
80 kVp	3.7 (3.3–4.8)	5.0 (4.0–5.0)	6.0 (5.0–6.0)	5.0 (4.0–6.0)
90 kVp	3.3 (2.7–4.0)	4.0 (3.5–4.5)	5.5 (5.0–6.0)	5.0 (3.0–6.0)
100 kVp	3.3 (2.7–4.0)	3.2 (3.0–4.5)	5.0 (4.0–5.5)	5.0 (3.0–5.0)
110 kVp	2.7 (2.3–3.8)	3.0 (2.3–4.0)	5.0 (4.0–5.0)	4.5 (3.0–5.0)
120 kVp	2.3 (1.7–3.8)	3.0 (2.3–4.0)	5.0 (3.0–5.0)	4.5 (3.0–5.0)
125 kVp	1.7 (1.2–3.5)	2.5 (2.3–4.0)	5.0 (3.0–5.0)	4.5 (3.0–5.0)

All values are given in median (range) of the three readers after calculation the mean of their readings. The highest mean for both image quality and sharpness was found for 70 kVp. The lowest bone overlay (i.e. the highest dark-field signal despite the opacity) was found for the lowest tube voltages, going along with the very high dark-field signal strength at these voltages.

Signal strength score for attenuation radiographs showed an inverse trend as dark-field signal with the highest signal at high tube voltages (Fig. 2). The highest bone signal and sharpness were found at low tube voltages (Table 2). Image quality was higher for higher tube voltages, however, a median of 4.5 (4 = good image quality) or higher was found even for low tube voltages. Despite the clear trends, no significant differences (Wilcoxon-signed rank test) for the signal strength at different voltages could be found.

**Table 2:** Results of the reader study examining attenuation radiographs.

	Lung signal	Bone overlay	Image quality	Sharpness
50 kVp	4.0 (3.0–4.5)	3.8 (3.0–4.0)	4.5 (3.0–6.0)	6.0 (5.5–6.0)
60 kVp	4.0 (3.0–5.5)	4.0 (3.0–4.5)	5.5 (3.0–6.0)	6.0 (6.0–6.0)
70 kVp	4.5 (3.0–6.0)	3.5 (3.0–5.0)	5.5 (3.0–6.0)	6.0 (5.8–6.0)
80 kVp	5.0 (3.0–6.0)	3.0 (3.0–5.5)	6.0 (3.0–6.0)	5.5 (5.0–5.8)
90 kVp	5.0 (3.3–6.0)	4.0 (3.0–5.5)	5.5 (4.0–6.0)	5.0 (4.0–5.8)
100 kVp	5.5 (4.3–6.0)	4.3 (3.0–6.0)	6.0 (5.0–6.0)	5.0 (4.0–5.8)
110 kVp	6.0 (5.3–6.0)	5.3 (2.0–6.0)	6.0 (6.0–6.0)	5.0 (3.0–5.5)
120 kVp	6.0 (5.3–6.0)	5.3 (2.0–6.0)	6.0 (6.0–6.0)	5.0 (3.0–5.5)
125 kVp	6.0 (5.3–6.0)	5.3 (2.0–6.0)	6.0 (5.6–6.0)	4.0 (3.0–5.0)

All values are given in median (range) of the three readers after calculation the mean of their readings. The highest subjective lung signal and subjective image quality were found for high tube voltages. The highest sharpness scores were found for low tube voltages with the highest bone signal score at 50 kVp. Despite the high subjective bone signal, the rating for bone overlay (meaning how well the lung is visible despite the bone overly) only showed small differences between the tube voltages, ranging from a mean of 3.8 (at 50 kVp) to 5.3 (at 110–125 kVp).

### Quantitative evaluation

**Figure 3:**
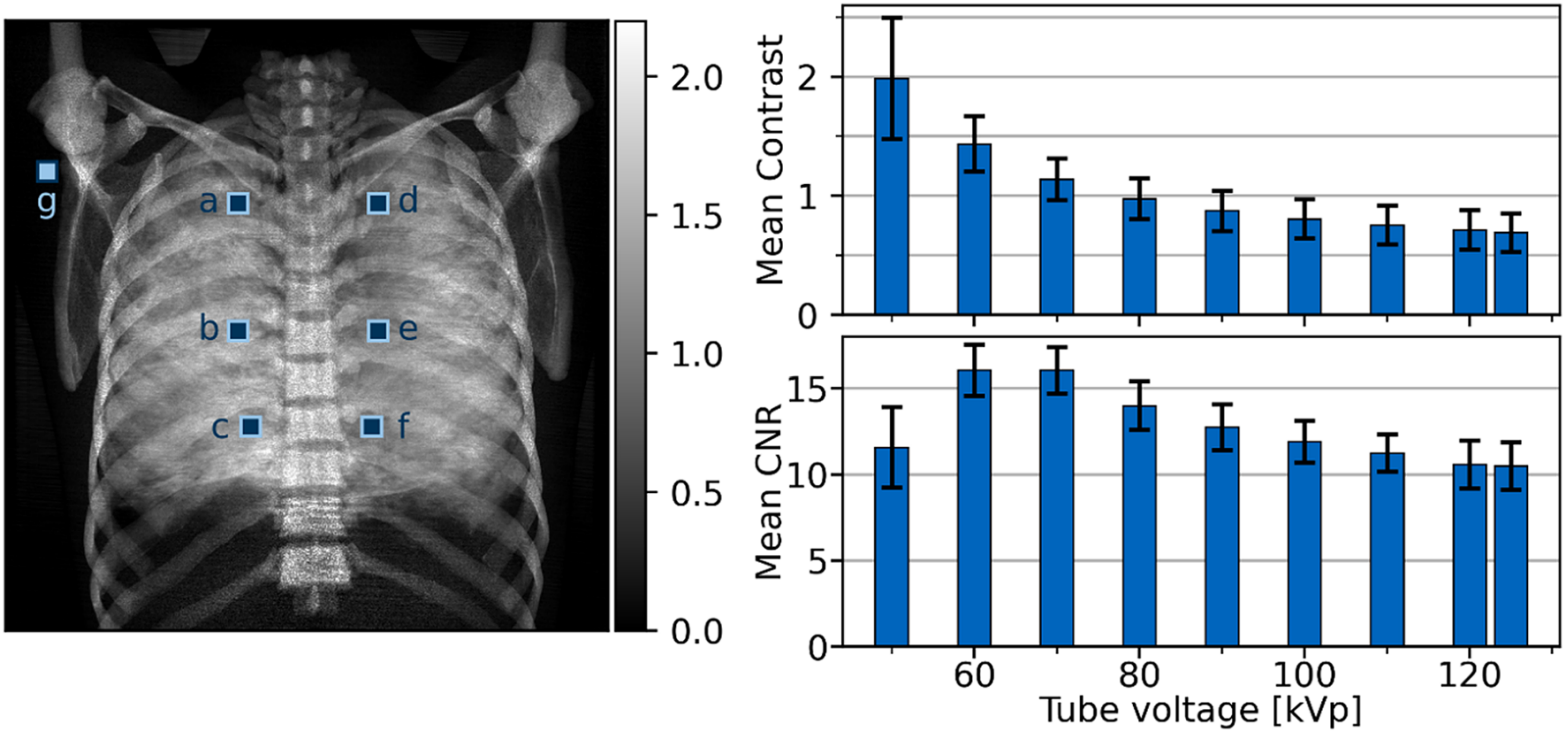
Left: Dark-field image of the Lungman, acquired with a tube voltage of 70 kVp. In the image position of the ROIs in the lung (**a**–**f**) and the position of the ROIs in the vicinity of the lung (**g**) are highlighted. Right: Mean contrast (top) and mean CNR (bottom) of the dark-field lung signal plotted for all examined tube voltages.

The mean dark-field contrast is depicted in Fig. 3. The contrast decreases towards higher energies. This is in accordance with the results from the reader study, where the dark-field signal strength score also decreases for higher energies. Even though contrast is highest for lower energies, the standard deviation (bars) for lower energies is higher than for higher energies. Looking at the mean contrast to noise ratio (CNR) in the lung one can see, that highest CNR is achieved at 60 to 70 kVp.

## Discussion

In the current study, dark-field images and attenuation radiographs obtained at different tube voltages were evaluated regarding subjective and objective image criteria. As in every examination a dark-field image and a corresponding attenuation radiograph are generated from the same data set, identical tube parameters for both modalities were guaranteed. Due to the simultaneous acquisition, a perfect spatial correlation between the two modalities is possible.

Quantitative evaluation of dark-field images showed that the highest signal strength was obtained at the lowest used tube voltage (50 kVp) and the highest CNR was obtained at 60 and 70 kVp. Subjective evaluation of dark-field images showed similar results with the highest subjective signal strength at 50 kVp. Achieving highest dark-field contrast and signal strength for low tube voltages was to be expected as dark-field signal decreases with the energy. However, despite a lower subjective dark-field signal strength, the highest image quality for dark-field images was reported for 70 and 80 kVp. At these voltages, a high subjective dark-field signal strength with a median value of 4.8 and 3.7 was still visible, respectively. Additionally, the highest sharpness was assigned at 50–70 kVp. Taking all the reported objective and subjective image parameters into account, a tube voltage of 70 kVp seems most favorable in the used setup. These reported differences were not statistically significant, which is most likely due to the low sampling number (3 readers and thus data points for each voltage as for repetitive measurements and lung areas mean values were calculated). For attenuation radiographs, the highest subjective image quality was found for 80 and 100–125 kVp. However, the median subjective image quality was still 5.5 for 70 kVp (5 indicating a very good image quality). As expected, a stronger opacity and thus overlay of bone was reported for low tube voltages in attenuation radiographs. Due to the decrease of the total interaction cross-section towards higher tube voltages for bone, the bones are less opaque at these energies compared to low energies. The total interaction cross-section for soft tissue also decreases towards higher energies. However, the decrease of the cross-section for bone is stronger than for soft tissue but both cross-sections will have similar values for high energies. Therefore, the bone signal strength (i.e. contrast) is lower for high tube voltages. Despite the higher overlay of bone, a high median subjective lung signal strength resulted for 70 (4.5) and 80 (5.0) kVp in attenuation radiographs, respectively. Thus, the artificial lung parenchyma was well visible despite the increased overlay of the bones at lower voltages. Obviously, the range of X-ray spectra yielding both attenuation radiographs and dark-field images with a high image quality is narrow: for attenuation radiographs, higher tube voltages are favorable (as used in clinical routine), whereas—at acceptable dose levels—for dark-field images lower tube voltages are needed.

Combining all results of the subjective and objective image evaluation, a tube voltage of 70 kVp seems to deliver an optimal dark-field image with a good attenuation radiograph at the same time. Hence, an evaluation of both modalities is possible at this tube voltage, making dark-field information available as well as the conventional attenuation radiograph including anatomical information during a single examination. The availability of both modalities at the same time is of special importance as different impairments of the lung can result in a reduced dark-field signal, e.g. emphysema and pneumonia: if the severity of emphysema is to be evaluated with dark-field imaging (which is clearly superior to attenuation imaging for this matter), one has to be sure that the decreased dark-field signal is not a result of a pneumonia, which can be excluded via the attenuation image.

The results of the current study confirm the results of a former study evaluating the inflated lung of a deceased body at different tube voltages^6^. In this study, a single body was examined, and the lung was imaged at subsequent time points. Additionally, the dose was not constant for the examined tube voltages. The described drawbacks could lead to distorted results, necessitating a standardized phantom study. The usage of a phantom in the current study enabled a standardized evaluation at the different tube voltages and it was taken care that all images were examined with the same radiation dose, expressed as the dose area product. Despite slightly different setups used in this study and in^6^ both yielded the same optimal tube voltage for dark-field examination at similar setups around 70 kVp as here, an ideal compromise between dark-field and attenuation radiography is present.

The current study has some limitations which have to be addressed. Although readers were experienced in dark-field imaging and received training before the beginning of the study, dark-field is not an established imaging technique used in clinical routine. This could lead to a misinterpretation by the readers. However, results of subjective and objective image criteria are consistent and earlier studies showed similar results, indicating a robust performance by the readers. The used phantom was a standardized lung phantom for conventional radiography. However, the originally present artificial lung tissue did not deliver a dark-field signal. Finding a suitable dark-field phantom material for the lung is difficult. Such a material should have the same microstructural shape and size, and the same electron density. However, even an ex-vivo lung does not resemble an in-vivo lung perfectly, as it starts to decompose and thus the microstructure of the lung is changed. As the main interest in this study was on the relative change of the dark-field signal with respect to different tube voltages and not the absolute dark-field signal strengths, it is sufficient for this study to use cotton wool as a phantom material. To reduce the influence of features from the cotton wool, only uncorrelated noise of the images was used for the CNR analysis. This uncorrelated noise corresponds not necessarily to the intrinsic image noise. However, it is a very good approximation. Furthermore, the amount of fatty tissue in the chest region of a patient might also influence the CNR and image quality of dark-field images. The phantom used in the current study models a person with a body-mass-index of 23 kg/m^2^. We do not expect a significant shift of the optimal tube voltage in terms of CNR and image quality if more fat or soft tissue is present and the tube current is adapted accordingly. Finally, the optimum tube voltage for dark-field is highly dependent on setup-specific parameters. Setups with longer inter grating distances or smaller grating periods might be able to produce good dark-field images at higher energies. Producing gratings with smaller periods but similar attenuating properties means producing gratings with higher aspect ratios. These are challenging to manufacture and therefore hard to come by. Increasing the setup length was also not deemed feasible, as the setup used in this study aims for a use in the setting of a typical clinical examination room, limiting the setup length. The current setup is planned to be used for imaging of patients, so this study was a milestone in preparation of the tube settings and definition of suitable imaging parameters for future dark-field imaging in humans.

In conclusion, the results of the current study will be highly important for future dark-field imaging of living humans. Before examining living human and thus causing additional radiation exposure, one has to ensure that the used imaging parameters are optimal for this matter. So far, only results and experiences of early animal studies and the imaging of dead humans exist, and these tests and studies were not performed using a system capable of examining living humans. As a matter of fact, repetitive imaging of living humans has to be avoided is not possible due to radiation exposure. Thus, the evaluation of the optimum tube voltage had to be performed using an anthropomorphic lung phantom. Using this information, we will be able to perform the worldwide first dark-field study for the imaging of living humans.

Considering the information and results generated in this study, the dark-field imaging system for the first examination of living humans was optimized and further in-vivo studies as well as clinical examinations can be performed. Hereby, a tube voltage of 70 kVp should be used to achieve both high quality attenuation radiographs and dark-field images.

## Materials

### Image acquisition

Images were acquired with a three grating Talbot-Lau interferometer with standard medical X-ray source (MRC 200 0508 ROT GS, Philips Medical Systems, Hamburg, Germany) and detector (Pixium 4343 F, Trixell, Moirans, France). The X-ray beam is filtered in total with 15.6 mm aluminum equivalent, measured at 70 kVp. Of those, a filtration of 2.7 mm aluminum equivalent was integrated in the X-ray tube and the remaining filtration of 12.9 mm aluminum equivalent originate from the interferometer. The effective field of view is $37 \times 37\;{\text{cm}}^{2}$ in the patient plane. A $3 \times 3$ pixel binning was used leading to an effective pixel size of $400 \times 400\;\mu {\text{m}}^{2}$ in the patient plane. Dark-field and attenuation images were acquired by using a modified version of the Moiré fringe scanning approach described in^25^. The attenuation image was processed with a similar algorithm as conventional radiographs employing histogram equalization and structural enhancement^26^. The dark-field signal $DF$ is calculated via $- {\text{ln}}\left( {\frac{{V_{s} }}{{V_{r} }}} \right)$, where $V_{s}$ is the sample and $V_{r}$ the reference visibility, i.e. the visibility achieved in an empty scan.

A standard human chest phantom for thoracic imaging, the Multipurpose Chest Phantom N1 “Lungman” (Kyoto Kagaku, Kyoto, Japan), was used for the imaging. However, it does not generate a dark-field signal in the lung in its original composition. Therefore, the original lung was removed, and the void was filled with cotton wool. Furthermore, the substitute bone material creates an increased dark-field signal compared to real bones.

Images were acquired at tube voltages of 60 to 120 kVp in 10 kVp steps and at 125 kVp (the maximal achievable tube voltage of the source). The tube current was adapted for each tube current so that the dose area product was almost constant at 30 µGym^2^. The scan time was kept constant for each scan.

### Reader study

Subjective image analysis was performed independently by three blinded radiologists with 4-, 11-, and 11-years of experience (F.M., D.P., A.A.F.). All readers were familiar with dark-field imaging due to their participation in multiple studies in the scientific field of dark-field imaging. As the Lungman phantom was not yet used in dark-field studies, the readers received a training before the start of the study to familiarize with this phantom. In this training session, images with low, moderate, and high signal strength were presented as a reference. Each reader performed two reading sessions with at least two weeks in between. In each reading session, all images from one modality (i.e. attenuation or dark-field) were shown simultaneously. Thus, a better comparability and identification of small differences were possible. Window settings were identical for all images of the respective modalities, transmission and dark-field. For each parameter, a six-point scale was used. The following image parameters were evaluated for attenuation and dark-field images: lung signal strength (1: low; 6: very high); image quality (1: not diagnostic, 2: sufficient, 3: satisfactory, 4: good, 5: very good, 6: excellent); sharpness, i.e. differentiation of adjacent structures (1: very high blurring, 2: high blurring, 3: medium blurring, 4: low blurring, 5: sharp edges, 6: very sharp edges), bone overlay, i.e. visibility of the lung in terms of bone overlay (1: high bone opacity/clearly impaired lung visibility, 6: low bone opacity/lung visibility not impaired). All parameters were evaluated for six areas of the phantom (left lung and right lung; upper zone, middle zone, lower zone).

### Statistical analysis

Statistical analysis was performed by dedicated software packages (SPSS 26, IBM, USA; Excel 2016, Microsoft, USA; Prism 8, Version 8.4.3, USA). For the reader study, the median of scores was assessed, and the means of reading sessions 1 and 2 were calculated for each parameter. The mean values of every reader and all lung areas were used to calculate the mean value for the evaluated parameters at each tube voltage. These mean values of each reader were used to calculate the median and range of the three readers for each parameter and each tube voltage. Statistical evaluation of subjective image criteria in the reader study was performed using Wilcoxon signed-rank test as pairwise comparisons of different tube voltages was performed. A *p*-value ≤ 0.05 was considered to indicate statistical significance.

### Quantitative evaluation of dark-field signal

Six regions of interest (ROIs), one in each lung zone, and one ROI in the vicinity of the lung (shoulder area) were selected for quantitative evaluation (Fig. 3). Each ROI has a size of $30 \times 30$ pixels corresponding to $12~ \times ~12\;{\text{mm}}^{2}$ in the patient/phantom plane. The size and position of the ROIs were selected such that ribs were excluded from the ROI and thus from the evaluation.

To reduce the influence of features from the cotton wool on the evaluation, a Laplacian Pyramid was used. Here, the images are separated into subbands, that contain the different frequency components of the image. The high frequency band (${L}_{0}$) contains uncorrelated noise and is therefore used for the noise characteristics of the dark-field images.

Mean values ${\mu }_{r,U}$ of the dark-field images and standard deviation ${\sigma }_{r,U}$ of the high frequency bands were calculated for each ROI $r$ at every tube voltage $U$ (for abbreviations also see Fig. 3):

$$\mu _{{r,U}} = \langle DF\left( U \right)\rangle_{r} ,~\quad ~\sigma _{{r,U}} = \sqrt {Var_{r} \left( {L_{0} \left( U \right)} \right)} ~\quad ~\left( {r = a,~ \ldots ,~g} \right).$$

Furthermore, the dark-field contrast $C_{{r,U}}$ between an ROI in the lung and the ROI in the vicinity of the lung and their standard deviations $\sigma _{{r,U}}^{{\left( C \right)}}$ were calculated using:

$$C_{{r,U}} ~ = ~\mu _{{r,U}} - \mu _{{g,U}} ,\quad \sigma _{{r,U}}^{{\left( C \right)}} = \sqrt {\sigma _{{r,U}}^{2} + \sigma _{{g,U}}^{2} } \quad \left( {r = a,~ \ldots .,f} \right).$$

From these values the contrast to noise ratio $CNR_{{r,U}}$ was calculated for each ROI in the lung:

$$CNR_{{r,U}} = ~\frac{{C_{{r,U}} }}{{\sigma _{{r,U}}^{{\left( C \right)}} ~}}\quad ~\left( {r = a,~ \ldots ,f} \right).$$
